# White-nose syndrome initiates a cascade of physiologic disturbances in the hibernating bat host

**DOI:** 10.1186/s12899-014-0010-4

**Published:** 2014-12-09

**Authors:** Michelle L Verant, Carol U Meteyer, John R Speakman, Paul M Cryan, Jeffrey M Lorch, David S Blehert

**Affiliations:** Department of Pathobiological Sciences, School of Veterinary Medicine, University of Wisconsin-Madison, 2015 Linden Dr., Madison, Wisconsin USA; US Geological Survey – National Wildlife Health Center, 6006 Schroeder Rd., Madison, Wisconsin USA; Institute of Biological and Environmental Sciences, University of Aberdeen, Aberdeen, Scotland UK; US Geological Survey – Fort Collins Science Center, 2150 Centre Ave. Building C, Fort Collins, Colorado USA; US Geological Survey – National Center, Environmental Health, 12201 Sunrise Valley Dr., Reston, Virginia USA

**Keywords:** White-nose syndrome, Bats, Doubly labeled water

## Abstract

**Background:**

The physiological effects of white-nose syndrome (WNS) in hibernating bats and ultimate causes of mortality from infection with *Pseudogymnoascus* (formerly *Geomyces*) *destructans* are not fully understood. Increased frequency of arousal from torpor described among hibernating bats with late-stage WNS is thought to accelerate depletion of fat reserves, but the physiological mechanisms that lead to these alterations in hibernation behavior have not been elucidated. We used the doubly labeled water (DLW) method and clinical chemistry to evaluate energy use, body composition changes, and blood chemistry perturbations in hibernating little brown bats (*Myotis lucifugus*) experimentally infected with *P. destructans* to better understand the physiological processes that underlie mortality from WNS.

**Results:**

These data indicated that fat energy utilization, as demonstrated by changes in body composition, was two-fold higher for bats with WNS compared to negative controls. These differences were apparent in early stages of infection when torpor-arousal patterns were equivalent between infected and non-infected animals, suggesting that *P. destructans* has complex physiological impacts on its host prior to onset of clinical signs indicative of late-stage infections. Additionally, bats with mild to moderate skin lesions associated with early-stage WNS demonstrated a chronic respiratory acidosis characterized by significantly elevated dissolved carbon dioxide, acidemia, and elevated bicarbonate. Potassium concentrations were also significantly higher among infected bats, but sodium, chloride, and other hydration parameters were equivalent to controls.

**Conclusions:**

Integrating these novel findings on the physiological changes that occur in early-stage WNS with those previously documented in late-stage infections, we propose a multi-stage disease progression model that mechanistically describes the pathologic and physiologic effects underlying mortality of WNS in hibernating bats. This model identifies testable hypotheses for better understanding this disease, knowledge that will be critical for defining effective disease mitigation strategies aimed at reducing morbidity and mortality that results from WNS.

**Electronic supplementary material:**

The online version of this article (doi:10.1186/s12899-014-0010-4) contains supplementary material, which is available to authorized users.

## Background

Since emergence of white-nose syndrome (WNS) in 2007, bat populations of eastern North America have declined precipitously due to disease-related mortality [1–3]. The causative agent of WNS is the fungus *Pseudogymnoascus* (formerly *Geomyces*) *destructans* [4–6], which erodes unfurred skin comprising wing membranes, muzzles, and ears of hibernating bats, inducing physiological perturbations, altered behavior, and death [7]. Although underlying causes for mortality from this invasive cutaneous mycosis remain unclear, proposed mechanisms include disruptions to vital homeostatic functions such as thermoregulation and water balance [8]. For example, water and electrolyte losses across the ulcerated wing epithelium have been proposed to cause hypotonic dehydration [9] and acid–base disturbances [10]. Consequently, alterations in behavior have been observed in infected bats, including increased frequency of arousal from torpor during hibernation [11,12] and unusual day flights during winter [3]. High metabolic demands of such activities [13–15] likely also contribute to mortality of bats prior to spring emergence by accelerating depletion of fat reserves. However, physiological data linking altered behavior to increased energy demands in bats with WNS are lacking.

The doubly labeled water (DLW) method is widely applicable to the study of energetics in relation to homeostasis, behavioral adaptations, and resource allocation in both animals and humans [16]. This method is based on dynamic flux of hydrogen and oxygen through the body and ability to measure these flux rates over a period of time using labeled isotopes, ^2^H and ^18^O [17]. Following administration of these exogenous isotopes, they equilibrate throughout the body water pool. The total body water volume (TBW) can then be estimated from the dilution spaces of the isotopes when introduced at known concentrations and serves as a valuable indicator of body composition (ratio of lean body mass to fat) [18]. Notably the DLW method has been used in temperate-zone insectivorous bats in the wild (*e.g.*, *Myotis lucifugus* [19] and *Eptesicus fuscus* [20]), but there are no published reports of this method being used in bats hibernating over a protracted time period (*i.e.*, months).

To evaluate proposed causes of mortality from WNS, we used the DLW method to quantify energy expenditure and changes in body composition of hibernating little brown bats (*M. lucifugus*) experimentally infected with *P. destructans* to test the hypothesis that WNS increases metabolic demands during hibernation. We predicted that infected bats would exhibit greater changes in body composition, specifically decreased proportion of fat mass, over the course of the experiment compared to negative control bats as a result of higher daily energy expenditures and fat utilization. To further characterize previously reported physiologic outcomes associated with WNS, we analyzed blood chemistries of all bats at the end of the experiment to assess acid–base balance, electrolytes, and hydration status.

## Results

### Infection status and torpor patterns

Of the 39 bats treated with conidia from *P. destructans*, 32 bats (14 male, 18 female) developed epidermal wing lesions characteristic of WNS by the end of the 98-d experiment. The majority of these bats (n = 30) had mild to moderate WNS (severity scores 1 or 2 with median score of 1), while the remaining two had moderate to severe WNS (severity scores 3 and 4). Sex had no effect on the probability that a bat developed WNS (Fisher’s Exact Test, p = 0.4075). All infected bats, including animals that did not develop detectable WNS by histology (n = 7), were PCR-positive for *P. destructans*; all bats in the control (non-infected) group were PCR-negative for the fungal pathogen. Four infected bats and five control bats died prior to the end of the experiment.

Average torpor bout duration for infected bats following DLW injection was 9.13 (2.31) d with average arousal duration of 54 (10) min. Average torpor bout duration for control bats following DLW injection was 8.52 (2.34) d with average arousal duration of 55 (11) min. Differences in torpor-arousal patterns of bats between treatment groups were not significant (torpor bout duration, p = 0.5337; arousal duration p = 0.6508).

### Blood chemistry

Blood chemistry parameters were analyzed for 27 infected bats (10 male, 17 female) and 11 control bats (6 male, 5 female) from which sufficient blood sample volumes were collected. One infected bat did not have skin lesions characteristic of WNS on histopathology, but there were no discernible differences across parameters when compared to infected bats with confirmed WNS. Additionally, we were unable to collect sufficient blood volume for analysis from the two bats with most advanced pathology (WNS severity scores 3 and 4). Thus, blood chemistry values presented herein for infected bats represent bats with mild WNS pathology (median severity score of 1).

**Figure 1:**
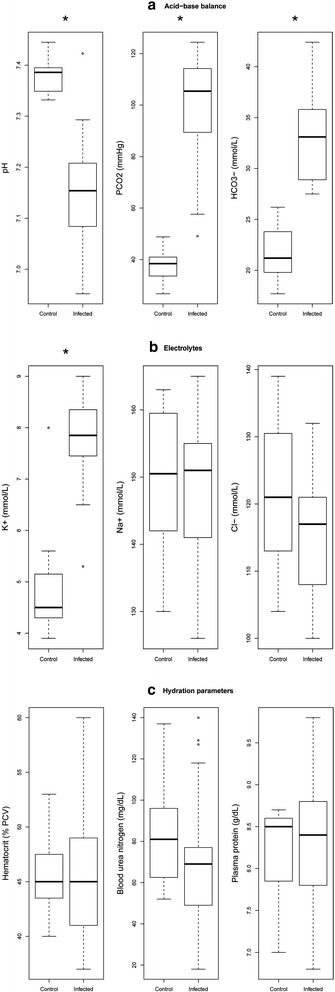
**Blood chemistry parameters.** Box-and-whisker plots of blood chemistry values for hibernating little brown bats (*Myotis lucifugus*) experimentally infected with *Pseudogymnoascus destructans* and negative (non-infected) controls. Parameters for acid-base balance **(a)**, electrolytes **(b)** and hydration status **(c)** are shown. The median (bold line), upper and lower quartiles (box), and maximum and minimum values (whiskers) are shown. Potential outliers (points) shown were not confirmed by Bonferonni outlier tests. Significant differences (*) were determined at α = 0.05 corrected for multiple comparisons by the Holm-Bonferonni method.

Infected bats had significantly lower blood pH than controls (Table 1, Figure 1a). This acidemia was associated with a significant elevation of pCO_2_ in infected bats compared to controls (Table 1, Figure 1a), indicating that bats had respiratory acidosis. Bicarbonate levels of infected bats were also significantly higher than those of non-infected controls (Table 1, Figure 1a), evident of a compensatory renal response to a chronic acidosis. The accumulation of bicarbonate in blood of infected bats was also reflected in an elevated base excess compared to controls (Table 1). There were no differences in sodium or chloride concentrations between treatment groups, but potassium concentration was significantly higher in the infected bats than it was in the controls (Table 1, Figure 1b). Glucose concentrations were lower in infected bats but not significantly (Table 1), and anion gap values were not different from controls nor elevated as would be expected if acidemia resulted from metabolic lactic or keto-acidosis. Other measures of hydration status (hematocrit, blood urea nitrogen, and total protein) were equivalent between treatment groups (Table 1, Figure 1c). Overall, there were no effects of sex (two-way MANOVA, Pillais’ Trace = 0.48, F (9,14) = 1.46, p = 0.25) or the interaction of sex and treatment (two-way MANOVA, Pillais’ Trace = 0.53, F (9,14) = 1.79 , p = 0.16) on measured blood parameters. See Additional file 1 for a complete table of blood chemistry parameter estimations compared to available reference values.

**Table 1:** **Blood chemistry comparisons**

**Parameter**	***k***	**df**	**Unadjusted p-value α = 0.05**	**Holm-Bonferroni corrected p-value (α/** ***k*** **)**
pCO_2_	12	38	<0.0001*	.0042
K	11	35	<0.0001*	.0045
pH	10	38	<0.0001*	.0050
HCO_3_^−^	9	36	<0.0001*	.0056
BE	8	37	<0.0001*	.0063
Glucose	7	26	.0144	.0071
AG	6	26	.0483	.0083
Cl^−^	5	22	.0646	.01
BUN	4	36	.2093	.0125
TP	3	36	.6085	.0167
Na^+^	2	36	.7079	.025
Hct	1	23	.8396	.05

*Abbreviations*: *pCO*_*2*_ dissolved carbon dioxide, *K* potassium, *HCO*_*3*_^*−*^ bicarbonate, *BE* base excess, *AG* anion gap, *Cl* ^*−*^ chloride, *BUN* blood urea nitrogen, *TP* total protein, *Na* ^*+*^ sodium, *Hct* hematocrit.

Results of *t*-tests used to compare mean blood chemistry parameters for little brown bats (*Myotis lucifugus*) either experimentally infected with *Pseudogymnoascus destructans* or negative (non-infected) controls. Significant differences between treatment groups (*) were determined by p-values < Holm-Bonferroni p-values corrected by the index of comparison (*k*).

### Measurements of daily energy expenditure and total body water

Upon collection of final blood samples, 67 d after injection with DLW, labeled isotope concentrations had decreased to levels statistically indistinguishable from background levels. Consequently, isotope turnover rates and daily energy expenditure (DEE) could not be determined for bats euthanized at the end of the experiment. However, one bat in the control group that died 35 d after initial injection of DLW had detectable isotope concentrations at time of death. For this bat, k_d_ and k_o_ were 0.004, calculated respiratory CO_2_ production (*r*CO_2_) was 0.01 ml/min, and resultant DEE was 0.44 kJ/day.

Measurements of total body water (TBW) were available for 26 bats (19 infected, 7 controls) following initial injection of DLW and for 24 bats (17 infected, 7 controls) after final injection (Table 2). The reduction in sample size is attributed to mortality of bats during the experiment or inability to collect a sufficient amount of blood for isotope analyses. At the time of initial DLW injection, mean TBW as percent of body mass (TBW % BM) was significantly lower for infected bats than controls (Tables 2 and 3), but mean body mass was not different between groups (t = −0.5925, df = 30, p = 0.558). After 67 d hibernation, changes in TBW and body mass were compared for infected and control bats for which paired measurements were available. Body mass decreased significantly in both treatment groups (infected: t = 11.32, df = 13, p <0.0001; control: t = 14.24, df = 3, p = 0.0008), but the loss in body mass was equal between groups. Changes in TBW % BM were marginally significant between infected and control bats, but not when corrected for multiple comparisons (Tables 2 and 3). Within groups, infected bats, which all developed mild WNS (n = 14; median WNS severity score 1, range 1–2) exhibited a significant increase in TBW % BM (Tables 2 and 3); increase in TBW % BM among control bats (n = 4) was not significant (Tables 2 and 3).

**Table 2:** **Body composition measurements**

**Parameter**	***Pd*** **infected bats**	**Control bats**
***All data***	***n = 22***	***n = 10***
Initial TBW % BM	53.4 (4.3)	58.8 (2.4)
Final TBW % BM	60.5 (3.9)	61.0 (4.6)
Initial BM (g)	7.64 (0.75)	7.48 (0.58)
Final BM (g)	6.60 (0.68)	6.41 (0.57)
*Paired data*	*n = 14*	*n = 4*
Change in TBW % BM	+ 9.1 (3.5)	+ 4.0 (3.9)
Fat energy use (kJ)	43.9 (13.6)	20.5 (14.7)

Values of body mass (BM) and total body water as percentage of body mass (TBW % BM) were used to estimate net fat energy use over 67 d for individual little brown bats (*Myotis lucifugus*) experimentally infected with *Pseudogymnoascus destructans* and negative control (non-infected) bats. Values in the table are mean (SD) and n = sample size. Initial TBW % BM was significantly different between treatment groups. Over the course of the experiment, infected bats demonstrated a significant increase in TBW % BM and used significantly more fat energy than non-infected bats. Body mass decreased significantly over the experiment in infected and control bats but there was no difference between groups.

**Table 3:** **Doubly labeled water comparisons**

**Measurement**	***k***	**df**	**Unadjusted p-value α = 0.05**	**Holm-Bonferroni corrected p-value (α/** ***k*** **)**
Fat energy use (I)	8	13	<0.0001*	0.0063
Change in TBW % BM (I)	7	13	<0.0001*	0.0071
Initial TBW % BM (I vs C)	6	25	0.0041*	0.0083
Fat energy use (I vs C)	5	16	0.0088*	0.01
Change in TBW % BM (I vs C)	4	16	0.0243	0.0125
Fat energy use (C)	3	3	0.0687	0.0167
Change in TBW % BM (C)	2	3	0.1353	0.025
Final TBW % BM (I vs C)	1	22	0.7841	0.05

Results of *t*-tests used to compare doubly labeled water measurements for little brown bats (*Myotis lucifugus*) either experimentally infected with *Pseudogymnoascus destructans* (I) or non-infected controls (C). Total body water is represented as percentage of body mass (TBW % BM). Significant differences between treatment groups (*) were determined by p-values < Holm-Bonferroni p-values corrected by the index of comparison (*k*).

As trends in TBW, namely TBW % BM, reflect changes in body composition, net fat energy utilization was calculated from the change in TBW (in g) and body mass measurements for all bats with paired data assuming constant 73% water content of lean mass (see Additional file 2). Based upon these calculations, mean total fat energy utilization for the 67 d after which bats were administered DLW was significantly higher for bats with WNS compared to negative controls (Tables 2 and 3).

## Discussion

**Figure 2:**
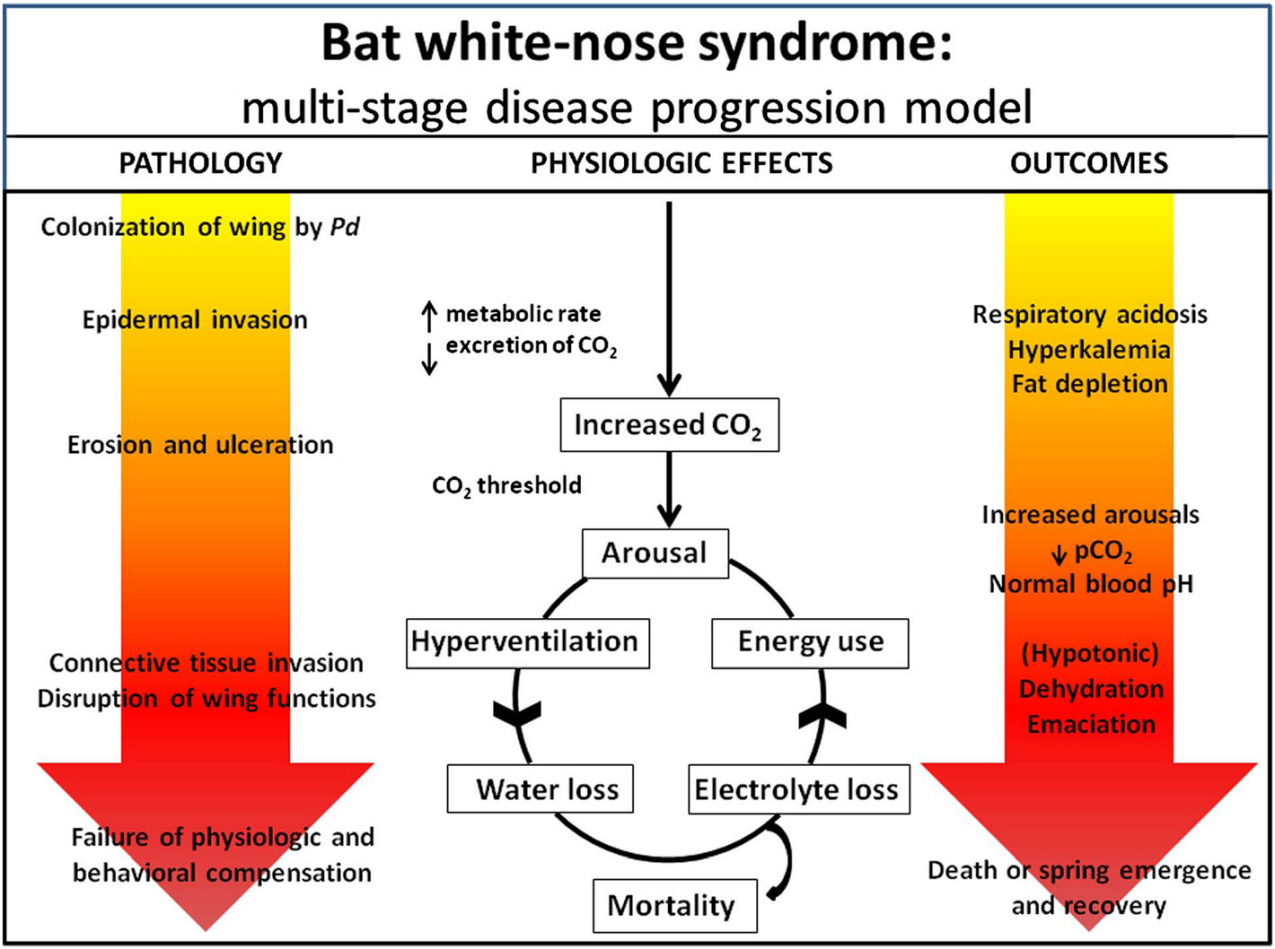
**Disease progression model for bat white-nose syndrome (WNS).** We propose a mechanistic multi-stage disease model for WNS in a hibernating bat that encompasses current knowledge on the progression of fungal-induced wing pathology and physiologic sequelae leading to mortality from disease. Initial colonization and invasion of the wing epidermis by *Pseudogymnoascus destructans* (*Pd*) results in increased energy expenditure, chronic respiratory acidosis (elevated blood pCO_2_ and bicarbonate), and hyperkalemia (elevated blood potassium). Erosion and ulceration of the epidermis stimulate increased frequencies of arousal from torpor, which remove excess CO_2_ and normalize blood pH, but contribute to dehydration and depletion of fat reserves. As wing pathology becomes more extensive and severe, these effects are exacerbated by water and electrolyte loss across the epidermis (hypotonic dehydration), which stimulate more frequent arousals and create a positive feedback loop that ultimately leads to mortality when energy reserves and compensatory mechanisms become exhausted.

Results of this study support the hypothesis that infection with *P. destructans* and subsequent development of WNS increases energy (fat) use in hibernating bats and provide key information for understanding the progression of physiologic disturbances that ultimately lead to mortality from this disease. Specifically, isotope-based estimates of changes in body composition provided evidence that hibernating little brown bats with WNS utilized twice as much energy as non-infected control bats housed under equivalent experimental conditions. However, the greater energy use by infected bats was not associated with an increased rate or duration of arousals from torpor. This implies that bats, even with mild WNS lesions, have an elevated metabolism prior to the onset of altered arousal patterns characteristic of late-stage infections [12]. Additionally, bats with early-stage WNS developed severe, chronic respiratory acidosis and hyperkalemia (high potassium concentrations in the blood). Integrating these results with those reported by others [7,9–12,21], we propose a mechanistic multi-stage disease progression model for WNS that encompasses our current knowledge of disease pathology and physiologic sequelae, including death, that result following infection by *P. destructans* (Figure 2).

As shown in this study, early stages of WNS involving fungal colonization of the wing membrane with progression to erosion and ulceration of the epidermis, are characterized by increased CO_2_ levels in blood, resultant acidemia, and hyperkalemia. The accumulation of CO_2_ may stem from either increased CO_2_ production from an elevated metabolic rate associated with infection, decreased CO_2_ expiration, possibly due to inhibition of diffusion across the damaged wing epithelium [7], and/or a compensatory response by the host to attempt to further lower torpor metabolic rates (and conserve fat reserves) by inducing a hypercapnic (high pCO_2_) acidosis [15,22–24]. This acidosis may then contribute to the observed hyperkalemia by an acidosis-induced extracellular shift of potassium. Intracellular potassium ions may also leak into the blood through damaged and necrotic cell membranes caused by hyphal invasion of the epidermis. Overall, these physiologic effects result in a chronic respiratory acidosis, hyperkalemia, and reduction of fat reserves among bats during early stages of WNS.

Consistent with results observed in bats at later and more severe stages of WNS [9,10], we propose that once pCO_2_ elevates beyond a tolerance threshold, chemoreceptors stimulate hyperventilation, and resulting increased arousals from torpor serve to remove excess CO_2_, returning blood pH to normal [22,25–28]. The high energy demand of these arousals then likely further contributes to accelerated depletion of fat reserves. Additionally, increased ventilatory rates and greater vapor pressure difference with increased body temperatures during arousals would contribute to greater evaporative water loss [29] and dehydration. Together, these outcomes are consistent with data previously published for little brown bats with more severe WNS pathology that exhibited increased frequency of arousal from torpor, decreased pCO_2_, normal blood pH, and dehydration [10].

As WNS progresses towards more extensive and severe wing lesions, dehydration may be further exacerbated by water and electrolyte loss across the damaged epidermis of the wing [9,30], further stimulating arousal from hibernation to drink [29,31,32]. Positive feedback loops are then established that link worsening disease-associated wing pathology to further increases in arousal frequency, water loss, and energy use resulting in additional observed acute physiologic changes, including hypocapnia, hypoglycemia, hyponatremia, hypochloremia, and emaciation [9–11]. Once compensatory mechanisms such as cellular buffering, respiratory and metabolic regulation, and/or behavioral adaptations are exhausted, this suite of disturbances ultimately leads to mortality unless the bat has sufficient energy reserves to persist until spring emergence and clear the infection following a return to a metabolically active state [33].

Although normal reference ranges for blood chemistry values in microchiropteran species are generally lacking [34], deviations of measured parameters in infected bats from the negative controls in this study, together with published information for apparently healthy hibernating little brown bats [10,34–39], suggest pathologic and potentially life-threatening physiological disturbances associated with early-stage WNS infections [see Additional file 1]. Bats with WNS in this study had almost 40% higher mean pCO_2_ than negative control bats (99.4 and 37.1 mmHg respectively), and pCO_2_ values above 90 mmHg are generally considered to be lethal in other non-hibernating animals and humans. Elevated pCO_2_ levels and associated acidemia are known to interfere with enzymatic functions, reduce metabolic activity, and cause central depression of respiration; in severe cases, such elevated levels can lead to coma and death.

Direct calculations of energy expenditure could not be determined for most bats in this study because final isotope concentrations were indistinguishable from background levels. However, the increase in TBW as a percent of body mass observed in infected bats indicated that bats with WNS had higher proportions of lean tissue mass to fat tissue mass at the end of the study. This finding implies that bats with WNS used significantly more fat energy reserves compared to negative controls despite hibernating under equivalent conditions. From the estimated changes in fat content over the 67-d measurement period, infected bats utilized 0.65 kJ/d, while control bats utilized 0.31 kJ/d. These results indicate that bats with WNS expended approximately twice as much energy during hibernation as non-infected control bats. Some caution must be taken in interpreting these results due to the small number of control bats for which paired TBW measurements were available to calculate estimates of fat use. However, the range of expected daily energy use of 0.27 to 0.51 kJ/d predicted for healthy hibernating *M. lucifugus* at the mean temperature of our study [40] is consistent with the daily rates of energy use observed in control bats in this experiment, and lower than rates observed in infected bats. There were no differences in torpor-arousal patterns between treatment groups in this study suggesting that WNS causes an increase in metabolism that is not directly associated with arousal from torpor and occurs at early stages in disease progression. Additionally, there were no differences between infected and control bats in T_skin_ maintained during torpor or arousal bouts. Alternatively, metabolic costs associated with infection and development of wing pathology may be linked to increased costs of thermoregulation caused by inhibition of peripheral vasoconstriction during torpor and arousal [25], catabolism of fat to generate metabolic water in response to increased water loss, or additional energetic costs associated with the host-pathogen interaction.

Rising concentrations of pCO_2_ in a mammal would normally stimulate increased respiration to release excess CO_2_. However, the unique physiology of mammalian hibernators allows for active suppression of respiration during torpor, and under these conditions, blood pCO_2_ increases to levels higher than those observed in metabolically active mammals [22,28,41,42]. This elevation of pCO_2_ is thought to be an integral part of hibernation physiology as induction of an acidotic state serves to reduce metabolic rate and thermogenesis [25,43,44]. Additionally, the resultant high pCO_2_ gradient improves ventilation efficiency, thereby minimizing energy costs of respiration during torpor [23]. Despite this tolerance for a respiratory acidosis [45], a hibernating mammal must still be able to regulate pCO_2_ for proper physiologic function. If CO_2_ elimination routes, such as passive diffusion of CO_2_ across the wing epithelium [46,47] are compromised by disease, as hypothesized for bats with WNS [7], persistently rising blood pCO_2_ levels would cause the severe chronic respiratory acidosis we have observed. Thus, underlying causes for the high pCO_2_ levels in bats with WNS are likely a combination of the uniquely adapted physiology of a hibernating mammal compounded by pathologically induced insult(s) to these physiological mechanisms.

Bat WNS presents a new paradigm for the study of infectious disease. Never before has a fungal skin pathogen been known to specifically infect a hibernating mammal, causing severe physiologic disturbances and mortality. Although substantial efforts have been devoted to understanding chytridiomycosis, a lethal fungal skin disease of amphibians, prior to WNS there had been no in-depth study of disease processes in a metabolically repressed animal caused by a psychrophilic and metabolically active pathogen. The poorly characterized capacity of bats to compensate for and respond to infection during hibernation demonstrates the difficulty of understanding how host-pathogen interactions influence disease manifestation and mortality.

## Conclusions

This study demonstrated that infection with *P. destructans* and subsequent development of WNS increased energy (fat) use in hibernating bats prior to the onset of altered arousal patterns associated with later stages of WNS. Severe, chronic respiratory acidosis and hyperkalemia were also apparent in bats that developed mild WNS. With these results, we present a multi-stage disease progression model for WNS as a framework for understanding the pathogenesis and underlying causes of mortality due to WNS (Figure 2). This model integrates a range of published work [7,9–12,21] with data from this study into the first attempt to mechanistically define a comprehensive conceptual model of what may occur during development of WNS in a hibernating bat – from initial colonization of wing skin by *P. destructans* until the death of the animal. This model identifies key testable hypotheses necessary to develop a comprehensive understanding of the physiologic effects of WNS on hibernating bats. Ultimately, this knowledge will be critical for guiding effective and properly timed management actions to moderate physiologic effects of WNS and minimize morbidity and mortality from this devastating disease.

## Methods

### Bats

This study was conducted in accordance with experimental protocol #110921 approved by the Institutional Animal Care and Use Committee of the USGS – National Wildlife Health Center (NWHC). Sixty (30 male and 30 female) little brown bats (*Myotis lucifugus*) were collected from a hibernaculum in Wisconsin on December 21, 2012 and transported to NWHC. Bats were held individually within tube socks maintained in coolers at approximately 7°C during transport. Body mass and right forearm length were recorded for each bat, and both wings of each bat were evaluated for pre-existing injuries. All animals were confirmed negative for *P. destructans* by polymerase chain reaction (PCR) [48] analysis of wing-skin swab samples (PurFlock nylon-flocked swabs, Puritan, Guilford, ME) collected from each bat. An archival temperature logger (iBBat, Alpha Mach) was affixed between the scapulae of each bat using a latex-based adhesive (Ostobond, M.O.C., Vaudreuil, Quebec, Canada) after trimming a 1 cm × 1 cm patch of fur to within 1 mm of the skin surface. Loggers were programmed to record skin temperature (T_skin_) every 15 minutes starting at the time of DLW injection until the end of the experiment. Torpor-arousal patterns were assessed using T_skin_ data downloaded from iBBat temperature loggers following termination of the experiment. Arousal thresholds were defined as 10% of maximum T_skin_ for each individual [11].

Bats were sorted by sex and randomly assigned to infected (n = 39; 19 males, 20 females) and control (n = 21; 11 males, 10 females) groups. Conidia of *P. destructans* (5 × 10^5^ in 20 μl of PBS with 0.5% Tween-20) were applied to the skin of the dorsal surface of both wings of each bat in the infected group as previously described [49]. PBS Tween-20 lacking conidia was similarly applied to the wings of control (non-infected) bats. Each group was placed into a mesh enclosure (22” H × 14” W × 14” D; Apogee Reptaria, Reptiledirect.com) within separate environmental incubators (Percival Scientific, Perry, IA) and maintained at 7.5°C and 90% RH for 98 days. Bats were monitored every other day for the duration of the experiment and any dead animals removed.

At the end of the experiment, all remaining bats were euthanized. Polymerase chain reaction (PCR) analyses of wing skin were performed as previously described [48] to confirm the presence of *P. destructans*. Additionally, the entire membrane of one wing was examined by histology to identify lesions diagnostic for WNS [21] and to assign severity scores [from 0 (no lesions) to 4 (severe, extensive lesions)] based on extent of fungal infection [11].

### Doubly labeled water

The DLW method used a two-sample approach by measuring isotopic concentrations at equilibrium (1 h following DLW injection) and once more at the end of the experiment [17]. The duration of the elimination period was determined from estimates of isotope washout rates modeled using parameters of energy expenditure for hibernating little brown bats [40], assuming torpor bouts of 8 to 16 d interspersed by 0.5 to 1.5 h arousals, as recorded for little brown bats with and without WNS in previous experiments [12]. One additional arousal and a 3-h euthermic period were included in the model to account for initial DLW injection and blood collection at the end of the experiment. Based upon an energy expenditure budget of approximately 60 kJ, isotope washout was predicted to occur 89 to 171 d post-administration of DLW. To increase the likelihood that labeled isotope concentrations would remain measurable over a duration of time sufficient for development of experimentally induced WNS (90 to 120 d; [12,49]), we administered the first dose of DLW 28 d after treatment of bats with fungal conidia.

For administration of DLW, bats were removed from incubators and aroused at room temperature for 20 to 30 min. Once fully aroused, both infected (n = 34) and negative control (n = 16) bats were injected intraperitoneally with approximately 70 μl of a mixture of enriched ^18^O (approximately 19 atom %) and ^2^H (approximately 11 atom %). Dose enrichments were quantified using a standard dilution experiment [50]. Bats were then held at room temperature for approximately 1 h to allow for isotopic equilibration [20]. Following 1 h at euthermic temperatures, 50 to 75 μl of blood was collected from the ventral aspect of the uropatagial vein of each bat [51] into two heparinized 100 μl capillary tubes for determination of initial isotope concentrations and TBW. Ends of the capillary tubes were immediately flame-sealed, dipped in sealing wax, and stored at 4°C until analysis. Bats were then returned to incubators and left undisturbed for the duration of the experiment. Additionally, five bats from each treatment group were used to measure background isotope levels [52]; these bats were treated identically as those described above, but they did not receive injections of DLW.

At 95 days post-treatment with conidia (67 d after injection of DLW) bats were removed from hibernation chambers, aroused to euthermic body temperatures, and blood was collected (as above) to determine final isotope concentrations. Blood from bats that did not receive initial DLW injections was also sampled at this time to obtain a concurrent measure of background isotope levels. Since TBW content of each bat was assumed to have changed since sampling at the beginning of the experiment, a dose of the DLW solution used previously was administered to each bat, for which a non-terminal blood sample was collected, to determine TBW at the end of the experiment for infected (n = 22) and control (n = 12) bats. Following the injection with DLW, bats were held at room temp for 1 h for isotopic equilibration, after which time they were anesthetized with 5% isoflurane and decapitated. Whole blood was collected into heparinized capillary tubes, and 50 to 75 μl of blood was immediately sealed in capillary tubes (as described above) for isotope analysis. Rectal temperature was recorded at the time of euthanasia using a miniature probe and digital thermometer (Models RET-4 & BAT7001H, Physitemp Instruments, Inc., Clifton, NJ).

### Blood chemistry

Following euthanasia, blood chemistry parameters were analyzed for each bat as previously described [9]. Briefly, 95 μl of whole blood was collected following decapitation and analyzed within one minute using an i-STAT portable clinical analyzer (EC8^+^ diagnostic cartridge, Abaxis, Union City, California, USA) to assess sodium (Na^+^, mmol l^−1^), potassium (K^+^, mmol l^−1^), chloride (Cl^−^, mmol l^−1^), pH, dissolved carbon dioxide (pCO_2_, mmHg), bicarbonate (HCO_3_^−^, mmol l^−1^), base excess (BE, mmol l^−1^), anion gap (AG, mmol l^−1^), blood urea nitrogen (BUN, mgdL^−1^), hematocrit (Hct, % PCV), and glucose (mgdL^−1^). Remaining blood was centrifuged in microtubes (Stat-Spin, Iris Sample Processing, Westwood, MA) for 90 s, and plasma protein (gdL^−1^) of the serum was measured using a hand-held refractometer (Pulse Instruments, Van Nuys, CA). Temperature-corrected values for pH and pCO_2_ were calculated using rectal temperature of the bat at the time of blood collection, and HCO_3_^−^, AG, and BE were then calculated using these temperature-corrected values according to the i-STAT manual [53] and specifications of the Clinical Laboratory Standards Institute for Blood Gas and pH Analysis and Related Measurements [54] (see also Additional file 3 for equations).

### Isotope analysis

Capillary tubes containing the blood samples were vacuum distilled [55], and water from the resulting distillate was used to produce CO_2_ and H_2_ (methods in [56] for CO_2_ and [57] for H_2_). The isotope ratios ^18^O: ^16^O and ^2^H: ^1^H were analyzed using gas source isotope ratio mass spectrometry (Optima, Micromass IRMS and Isochrom μG, Manchester, UK). Samples were run alongside three lab standards for each isotope (calibrated to International standards) to correct delta values to ppm. Isotope enrichments were converted to values of daily energy expenditure using a single pool model as recommended for this size of animal [58].

Dilution spaces for oxygen (N_0_) and hydrogen (N_d_) were calculated by the plateau method (Speakman and Krol, [59]). CO_2_ production was calculated using equation 7.17 of Speakman [50] and used to estimate DEE according to the Weir equation [59] (see Additional file 2 for details).

### Statistical analyses

Normality of measured parameters was assessed visually using histograms and Q-Q plots for each parameter. Assumptions of normality and homogeneity of variance were satisfied so no transformations were performed. Mean values of measured parameters for infected bats were compared to control bats using independent two-sample *t*-tests. Paired data sets from TBW estimates were compared using paired *t*-tests. All *t*-tests were two-tailed with α = 0.05, and critical values for significance were adjusted to control the family-wise error rate using the Holm-Bonferonni method [60] applied separately to each data set: blood chemistry parameters (12 comparisons), DLW measurements (8 comparisons), and torpor profiles (2 comparisons). All statistical analyses were conducted in *R* [61]. Data in the text are presented as means (SD).

## Additional files

Additional file 1:
**Table of Blood Chemistry Parameters.** Comparisons of blood chemistry parameter values for hibernating little brown bats (*Myotis lucifugus*) experimentally infected with *Pseudogymnoascus destructans* (*Pd*) and negative (non-infected) controls to available reference values. Additional file 2:
**Doubly Labeled Water.** Mean isotope enrichments of administered DLW formulations and background isotope concentrations in bats at the start and end of the experiment. Equations used for calculation of daily energy requirements and fat energy use. Additional file 3:
**Blood Chemistry Calculations.** Equations used to calculate temperature-corrected acid–base blood parameters.
