# Supplementary material for: White-nose syndrome initiates a cascade of physiologic disturbances in the hibernating bat host
Source: BMC Physiol. 2014 Dec 9;14:10. doi: 10.1186/s12899-014-0010-4 (PMC4278231; doi:10.1186/s12899-014-0010-4)
Supplement: Additional file 1: — Table of Blood Chemistry Parameters. Comparisons of blood chemistry parameter values for hibernating little brown bats (Myotis lucifugus) experimentally infected with Pseudogymnoascus destructans (Pd) and negative (non-infected) controls to available reference values. [file 12899_2014_10_MOESM1_ESM.pdf]

Additional file 1: Table of blood chemistry parameters

Comparison blood chemistry parameter values (95% confidence intervals) for hibernating little brown bats (*Myotis lucifugus*) experimentally infected with *Pseudogymnoascus destructans* (*Pd*) and negative (non-infected) controls to standard reference ranges (venous blood) and values published for healthy hibernating little brown bats prior to white-nose syndrome.

Parameter	Units	*Pd* Infected bats (n~28)	Control bats (n~11)	Mammalian reference range[a]	Published value for healthy hibernating *M. lucifugus*
pH		7.11 – 7.17	7.36 – 7.40	7.31 - 7.41	7.56[b]
pCO_2	mmHg	94.3 – 108.2	33.3 – 40.9	41 – 51	38[b]
HCO^{3}-	mmol/L	30.7 – 35.3	20.1 – 23.5	23 – 28	35[b]
AG	mmol/L	3.57 – 8.93	8.47 – 13.83	10 – 20	
BE	mmol/L	2.1 – 7.2	-4.9 – (-1.6)	-2 to 3	
Na^{+}	mmol/L	146 – 152	143 - 156	138 - 146	150[b]
Cl^{-}	mmol/L	112 – 119	115 – 128	98 - 109	
K^{+}	mmol/L	7.5 – 8.1	4.3 – 5.5	3.5 – 4.9	6.4 – 7.2[c] (serum)
Glucose	mg/dL	107 – 148	144 – 217	70 - 105	5 – 110[b,d]
Hct	% PCV	44 – 49	44 – 48	38 – 51	43 – 55[b,c,d,e,f]
BUN	mg/dL	57 – 82	68 – 100	17 – 56	20[d]
TP	g/dL	8.03 – 8.63	7.79 – 8.53	6 – 8.3	

Abbreviations: pCO_2 (dissolved carbon dioxide); HCO^{3}- (bicarbonate); AG (anion gap); BE (base excess); Na+ (sodium); Cl- (chloride); K+ (potassium); Hct (hematocrit); BUN (blood urea nitrogen); TP (total protein).

Sources:

a) Abbott Point of Care Inc: *iStat Instruction Manual.* Abbott Park, IL: Abbott Point of Care Inc; 2008.

b) Warnecke L, Turner JM, Bollinger TK, Misra V, Cryan PM, Blehert DS, Wibbelt G, Willis CKR: **Pathophysiology of white-nose syndrome in bats: a mechanistic model linking wing damage to mortality.** *Biol Lett* 2013, **9:**20130177.

c) Riedesel, ML and GE Folk, Jr. **Serum electrolyte levels in hibernating mammals**. *The American Naturalis* 1958, **92**: 307-312.

d) Kallen, FC. (1960). **Plasma and blood volumes in the little brown bat.** *American Journal of Physiology* 1960, **190**: 999-1005.

e) Riedesel, ML. **Serum magnesium levels in mammalian hibernators**. *Transactions of the Kansas Academy of Science* 1957, **60:** 99-141.

f) Blood, FR and CL Dodgen. **Energy sources in the bat**. *American Journal of Physiology* 1956, **187**: 151-154.
