# Supplementary material for: White-nose syndrome initiates a cascade of physiologic disturbances in the hibernating bat host
Source: BMC Physiol. 2014 Dec 9;14:10. doi: 10.1186/s12899-014-0010-4 (PMC4278231; doi:10.1186/s12899-014-0010-4)
Supplement: Additional file 2: — Doubly Labeled Water. Mean isotope enrichments of administered DLW formulations and background isotope concentrations in bats at the start and end of the experiment. Equations used for calculation of daily energy requirements and fat energy use. [file 12899_2014_10_MOESM2_ESM.pdf]

Additional file 2: Doubly labeled water

Isotope enrichments and background isotope concentrations

All values reported as mean (SD). Background isotope concentrations at the time of initial injection were 156.82 (0.61) ppm for ^{2}H and 1989.55 (1.69) ppm for ^{18}O (n = 10). For all bats injected with DLW (n = 50), initial mean enrichments were 1868.4 (230.5) ppm ^{2}H and 5101.1 (402.5) ppm ^{18}O. At the time of final blood collection, background concentrations were 154.8 (2.4) ppm ^{2}H and 1990.5 (1.8) ppm ^{18}O. Following the second DLW injection for dilution space determination at the end of the experiment in 34 bats, mean enrichments were 1841.8 (298.9) ppm ^{2}H and 4994.1 (740.8) ppm ^{18}O.

Equations used for calculations of daily energy requirements (DEE)

Dilution spaces for oxygen (N_0) and hydrogen (N_d) were calculated by the plateau method [60] because bat activity during the isotopic equilibrium period (1 h at euthermic body temperatures) differed from activity during the measured elimination period when the bats were primarily in torpor.

CO_2 production was calculated using Equation 7.17 [54], which is based on a single-pool model and accounts for isotopic fractionation during phase changes:

$$rCO_2 = (N/2.078) \text{ x } (k_o – k_d) – 0.0062\ k_d N,$$

where rCO_2 (mol d^{-1}) is the CO_2 production, N (mol) is the body water pool estimated by the dilution space for the oxygen isotope, and k_o and k_d (d^{-1}) are the respective turnover rates of the oxygen and hydrogen isotopes. This model, which assumes an equivalent dilution space for hydrogen and oxygen, has been validated as an accurate estimation technique for small mammals [60], including bats [22].

Daily energy expenditure (DEE) was then calculated using CO_2 production estimates and an assumed respiratory quotient (RQ) of 0.85 according to the Weir equation [62].

Calculation of fat energy reserves and utilization from TBW

The change in TBW between initial and final measurements for bats with paired data was used to calculate fat energy utilization according to body water distribution rules which state that lean tissue contains 73% water and is distinct from fat, which is anhydrous. Given that 97% of body mass is available for water exchange, gram measurements of body mass (BMg) and total body water (TBWg) can be used to calculate grams of fat tissue (FTg) by the equation:

$$FTg = (0.97*BMg)-(TBWg/0.73).$$

In terms of energy, fat tissue contains 39 kJ/gram, so total fat energy (kJ) = FTg*39.
