# Supplementary material for: White-nose syndrome initiates a cascade of physiologic disturbances in the hibernating bat host
Source: BMC Physiol. 2014 Dec 9;14:10. doi: 10.1186/s12899-014-0010-4 (PMC4278231; doi:10.1186/s12899-014-0010-4)
Supplement: Additional file 3: — Blood Chemistry Calculations. Equations used to calculate temperature-corrected acid–base blood parameters. [file 12899_2014_10_MOESM3_ESM.pdf]

Additional file 3: Blood chemistry calculations

Acid-base blood parameters (pH, pCO_2, HCO_3^-, BE, and Anion Gap) are temperature-dependent so values measured by the iSTAT at 37 °C (X_{37}) were corrected by the rectal temperature of the bat at the time of blood sample collection (T). The following equations were used to calculate temperature-corrected values (X_T):

1 $pH_T = pH_{37} - (T - 37)[\ 0.0146 + 0.0065(pH_{37} - 7.4)]$

2 $pCO_{2[T]} = pCO_{2[37]} \text{ x } 10^{[0.019(T - 37)]}$

3 $HCO_3^- = 10^{\ pH + \log10(PCO2) - 7.608}$

4 $BE = (HCO_3^- - 24.98) + [16.2 \text{ x } (pH - 7.4)]$

5 $\text{Anion Gap} = (Na^+ + K^+) - (Cl^- + HCO_3^-)$
